# Histone Modification and Chromatin Remodeling During the Seed Life Cycle

**DOI:** 10.3389/fpls.2022.865361

**Published:** 2022-04-25

**Authors:** Xiali Ding, Xuhui Jia, Yong Xiang, Wenhui Jiang

**Affiliations:** ^1^Guangdong Laboratory for Lingnan Modern Agriculture, Genome Analysis Laboratory of the Ministry of Agriculture, Agricultural Genomics Institute at Shenzhen, Chinese Academy of Agricultural Sciences (CAAS), Shenzhen, China; ^2^College of Life Science and Technology, Guangxi University, Nanning, China

**Keywords:** histone modification, chromatin remodeling, seed development, seed dormancy, seed germination, seedling establishment

## Abstract

Seeds are essential for the reproduction and dispersion of spermatophytes. The seed life cycle from seed development to seedling establishment proceeds through a series of defined stages regulated by distinctive physiological and biochemical mechanisms. The role of histone modification and chromatin remodeling in seed behavior has been intensively studied in recent years. In this review, we summarize progress in elucidating the regulatory network of these two kinds of epigenetic regulation during the seed life cycle, especially in two model plants, rice and Arabidopsis. Particular emphasis is placed on epigenetic effects on primary tissue formation (e.g., the organized development of embryo and endosperm), pivotal downstream gene expression (e.g., transcription of *DOG1* in seed dormancy and repression of seed maturation genes in seed-to-seedling transition), and environmental responses (e.g., seed germination in response to different environmental cues). Future prospects for understanding of intricate interplay of epigenetic pathways and the epigenetic mechanisms in other commercial species are also proposed.

## Introduction

Well-developed seeds assure species dispersion of parent plants and serve as important sources of human food. Progression from seed development to seedling establishment is the crucial phase of ontogenesis in spermatophytes. It involves a series of sequential physio-morphological state changes and includes several biological stages. The process starts with seed development and followed by maturation, when seed desiccation and seed dormancy are achieved in some species. After that, seeds germinate in a suitable environment, which marks the initiation of seedling establishment.

Proper seed development is inseparable from the organized establishment of all tissues (such as embryo, endosperm and seed coat), which is coordinated by changes in hormone levels and gene expression (Zhou et al., 2013; Figueiredo and Köhler, 2018). Seed maturation proteins in the LAFL regulatory network—LEAFY COTYLEDON 1 (LEC1), ABSCISIC ACID INSENSITIVE3 (ABI3), FUSCA3 (FUS3), and LEAFY COTYLEDON 2 (LEC2)—play predominant roles in triggering and maintaining embryonic cell fate by fine-tuning the expression of genes involved in the accumulation of storage protein and lipid reserves in the embryo (Giraudat et al., 1992; Keith et al., 1994; Lotan et al., 1998; Stone et al., 2001; Yamamoto et al., 2009; Lepiniec et al., 2018).

In some species, during the maturation stage of seed development, dormancy gradually increases, peaking in freshly matured seeds. Dormancy enables seeds to adapt to the environment and plants to maintain reproduction (Née et al., 2017). *DELAY OF GERMINATION1* (*DOG1*) is the master regulator of primary dormancy in Arabidopsis (*Arabidopsis thaliana*); the encoded protein is a temperature detector that directs dormancy cycling in response to seasonal changes (Alonso-Blanco et al., 2003; Bentsink et al., 2006; Nakabayashi et al., 2012; Graeber et al., 2013; Footitt et al., 2015).

In appropriate condition, seeds can germinate once exposed to water. Germination behaviors, including germination rate and efficiency, differ between species and varieties in response to environment cues or abiotic stress. These differences are mediated mainly through the antagonistic roles of the plant hormones gibberellic acid (GA) and abscisic acid (ABA) (Jacobsen et al., 2002; Oh et al., 2007; Holdsworth et al., 2008; Seo et al., 2009; Vaistij et al., 2013; Shu et al., 2016).

Seed germination marks the initiation of the seed-to-seedling developmental transition. In this process, the sources of seedling nutrition and energy acquisition gradually transition from consumption of seed storage substances to photoautotrophy, in conjunction with significant alteration of biosynthetic and signaling pathways (Zanten et al., 2013; Jia et al., 2014). Correspondingly, suppression of seed maturation genes, the LAFL, and activation of those involved in vegetative growth is indispensable to avoid ectopic proliferation of embryonic tissues and thus maintain the normal vegetative morphology of seedlings (Parcy and Giraudat, 1997; Lotan et al., 1998; Stone et al., 2001; Gazzarrini et al., 2004; Braybrook et al., 2006; Yang et al., 2013).

The different stages of the seed life cycle are not isolated and are each under precise control. Accurate DNA processing and subsequent gene transcript levels are tightly linked to chromatin status, which is regulated by epigenetic modification. Epigenetic changes, including DNA methylation, histone modifications, chromatin remodeling, and the activities of small RNAs, affect plants in many ways (Goldberg et al., 2007). Research on the effects of epigenetic regulation in seed biology has recently increased. This review focuses mainly on the effects of two types of key regulatory factors, histone modifiers and chromatin remodelers, on the pivotal phase of the seed life cycle (Table 1).

**TABLE 1:** List of histone modifiers, chromatin remodelers and associated regulators involved in seed life cycle.

Gene Name	Species	Locus	Seed development	Seed dormancy	Seed germination	Seedling establishment	References
HD2A	*A. thaliana*	AT3G44750	√		√		Wu et al., 2000; Colville et al., 2011
HD2C	*A. thaliana*	AT5G03740			√		Colville et al., 2011; Luo et al., 2012
HDA7	*A. thaliana*	AT5G35600	√				Cigliano et al., 2013
HDA9	*A. thaliana*	AT3G44680			√		Baek et al., 2020
HDA6	*A. thaliana*	AT5G63110			√	√	Tanaka et al., 2008; Chen and Wu, 2010; Chen L. T. et al., 2010; Luo et al., 2012
HDA19	*A. thaliana*	AT4G38130		√	√		Tanaka et al., 2008; Chen and Wu, 2010; Zhou et al., 2020
HDA15	*A. thaliana*	AT3G18520			√		Gu et al., 2017
ZmHDA108	Z. mays	GRMZM2G136067	√				Forestan et al., 2018
OsHDA705	*O. sativa L.*	Os08g25570			√		Zhao et al., 2016
OsHDT701	*O. sativa L.*	Os05g51830			√		Zhao et al., 2014
OsSRT1	*O. sativa L.*	LOC_Os04g20270	√				Zhang et al., 2016
OsGW6a	*O. sativa L.*	LOC_Os06g44100	√				Song et al., 2015
EFS	*A. thaliana*	AT1G77300	√		√	√	Tang et al., 2012; Lee et al., 2014; Cheng et al., 2018
SUVH5	*A. thaliana*	AT2G35160		√	√		Gu et al., 2019; Zhou et al., 2020
SUVH4	*A. thaliana*	AT5G13960		√	√		Zheng et al., 2012
ULT1	*A. thaliana*	AT4G28190				√	Xu et al., 2018
ATX1	*A. thaliana*	AT1G66240				√	Xu et al., 2018
LDL1, LDL2	*A. thaliana*	AT1G62830, AT3G13682		√			Zhao et al., 2015
REF6	*A. thaliana*	AT3G48430		√			Chen H. et al., 2020
JMJ20, JMJ22	*A. thaliana*	AT5G63080, AT5G06550			√		Cho et al., 2012
JMJ17	*A. thaliana*	AT1G63490			√		Wang et al., 2021
HUB1, HUB2	*A. thaliana*	AT2G44950, AT1G55250		√			Liu et al., 2007; Liu et al., 2011
MEA	*A. thaliana*	AT1G02580	√				Yadegari et al., 2000; Köhler et al., 2003b; Makarevich et al., 2006
FIE	*A. thaliana*	AT3G20740	√			√	Ohad et al., 1999; Yadegari et al., 2000; Wang et al., 2006; Bouyer et al., 2011
FIS2	*A. thaliana*	AT2G35670	√				Chaudhury et al., 1997; Hehenberger et al., 2012
MSI1	*A. thaliana*	AT5G58230	√				Köhler et al., 2003a
SWN	*A. thaliana*	AT4G02020	√			√	Chanvivattana et al., 2004; Schubert et al., 2005; Wang et al., 2006; Makarevich et al., 2006; Yang et al., 2013
CLF	*A. thaliana*	AT2G23380	√	√		√	Schubert et al., 2005; Makarevich et al., 2006; Yang et al., 2013; Liu J. et al., 2016; Chen N. et al., 2020
VRN2	*A. thaliana*	AT4G16845				√	Schubert et al., 2005
EMF2	*A. thaliana*	AT5G51230				√	Moon et al., 2003; Schubert et al., 2005; Tang et al., 2012;
OsFIE2	*O. sativa L.*	LOC_Os08g04270	√	√			Luo et al., 2009; Nallamilli et al., 2013; Li et al., 2014; Liu X. et al., 2016; Cheng et al., 2020;
OsFIE1	*O. sativa L.*	LOC_Os08g04290	√	√			Luo et al., 2009; Folsom et al., 2014; Huang et al., 2016; Cheng et al., 2020
OsSDG711	*O. sativa L.*	LOC_Os06g16390	√				Liu et al., 2021
OsEMF2a	*O. sativa L.*	LOC_Os04g08034	√				Luo et al., 2009; Tonosaki et al., 2021; Cheng et al., 2021
AtBMI1a, AtBMI1b, AtBMI1c	*A. thaliana*	AT2G30580, AT1G06770, AT3G23060				√	Bratzel et al., 2010; Chen D. et al., 2010; Yang et al., 2013
AtRING1a, AtRING1b	*A. thaliana*	AT5G44280, AT1G03770				√	Bratzel et al., 2010; Chen D. et al., 2010
EMF1	*A. thaliana*	AT5G11530				√	Moon et al., 2003; Kim et al., 2012; Xu et al., 2018
PKL	*A. thaliana*	AT2G25170		√		√	Ogas et al., 1997; Ogas et al., 1999; Dean Rider et al., 2003; Henderson et al., 2004; Li et al., 2005; Carter et al., 2018; Zha et al., 2020
BRM	*A. thaliana*	AT2G46020			√		Han et al., 2012
SWI3B	*A. thaliana*	AT2G33610			√		Saez et al., 2008
CHR12, CHR23	*A. thaliana*	AT3G06010, AT5G19310			√		Leeggangers et al., 2015
EBS	*A. thaliana*	AT4G22140		√	√		Narro-Diego et al., 2017; Li et al., 2020
S2Lb	*A. thaliana*	AT5G66240	√	√			Fiorucci et al., 2019
HDC1	*A. thaliana*	AT5G08450			√		Perrella et al., 2013
PWR	*A. thaliana*	AT3G52250			√		Yang et al., 2019
VAL1, VAL2	*A. thaliana*	AT2G30470, AT4G32010		√		√	Suzuki et al., 2007; Yang et al., 2013; Chen N. et al., 2020

Histone modifications, which are usually added to the N-terminus of the histone protein tail, can either regulate chromatin state directly or act as hotspots for the recruitment of other effectors to chromatin. Different histone modifications, including acetylation, methylation, ubiquitylation, and phosphorylation, comprise a “histone code” that provides a flexible method of governing gene transcription in response to developmental or environmental cues (Strahl and Allis, 2000; Turner, 2000; Dutnall, 2003). Histone modifications are reversible, being added and removed by “writer” and “eraser” enzyme complexes, respectively, that execute distinct functions on chromatin, promoting either active transcription or gene silencing (Kumar et al., 2021). For example, trimethylation of histone H3 lysine 4 (H3K4me3) and 36 (H3K36me3) are usually associated with gene activation, whereas H3K27me3, which is directly regulated by the classical polycomb repressive complex 2 (PRC2), correlates with heterochromatinization and transcriptional silencing (Berger, 2007; Zhang et al., 2007a,b, 2009; Mozgova and Hennig, 2015; Liu et al., 2019).

ATP-dependent chromatin remodeling complexes, with members of DNA-dependent ATPases as core subunits, utilize energy from ATP hydrolysis to disrupt the contacts between histones and DNA, thereby regulating dynamic access to packaged DNA. They mediate DNA replication, damage repair, and gene expression by changing the positions and occupancy of nucleosomes, introducing histone variants, and cooperating with histone-modifying factors (Goldberg et al., 2007; Hargreaves and Crabtree, 2011; Li et al., 2016). In eukaryotes, different epigenetic regulations often work coordinately to achieve cooperative or antagonistic modes of regulation (Li et al., 2015).

In this review, we summarize the histone modification and chromatin remodeling that occur during the seed life cycle, from seed development to seedling establishment. We hope to thereby pave the way toward a fundamental understanding and integration of the complex networks of epigenetic regulation acting in seed biology.

## Epigenetic Regulation of Seed Development

**FIGURE 1:**
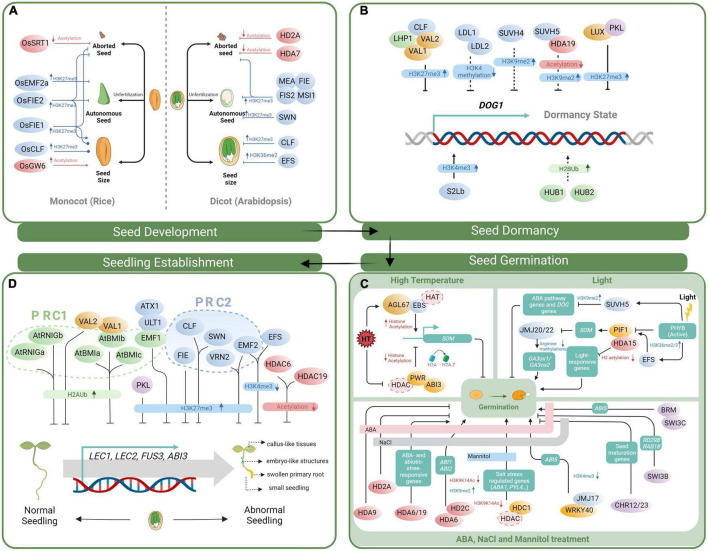
Model of histone modification and chromatin remodeling during various stages of the seed life cycle. **(A)** Epigenetic regulators act of pivotal seed development in Arabidopsis and rice. Line with a dot in tail end represent an unclear regulatory pathway. **(B)** Chromatin regulation of seed dormancy through mediating the expression of *DOG1*. Solid lines represent regulator that can modulate *DOG1* expression directly. Dash lines represent those regulatory mechanisms that are still unclear. **(C)** The roles of histone modifiers and chromatin remodelers in regulating seed germination in response to light, temperature and abiotic stress. In the box below, the bold line with pink, gray and blue colors represent ABA, NaCl and mannitol treatment, respectively. Ellipse with dotted outline represents that the specific regulator is not clear. **(D)** Epigenetic factors act in seed-to-seedling transition. Illustrated histone modifiers and chromatin remodelers act in repression of ectopic expression of LAFL genes, thereby maintaining normal vegetative morphology of seedlings. The histone modifiers for histone acetylation, methylation, and ubiquitination are marked in red, blue and green, respectively. Chromatin remodelers and epigenetic associated regulators are marked in purple and orange, respectively. Downstream genes are shown in boxes with aquamarine blue.

The production of viable seed is important for plant dispersal, and is a major focus of crop breeders because of its direct association with grain yield. Seed development involves the sequential and orderly formation of various structures, including the embryo, endosperm, and seed coat. Among angiosperms, monocotyledonous and dicotyledonous plants show both similarities and differences in seed development (Zhou et al., 2013). In this section, we primarily discuss epigenetic effects on seed formation in Arabidopsis and rice (*Oryza sativa*), the model dicot and monocot species, respectively (Figure 1A).

Histone deacetylases (HDACs) control seed-setting rate through affecting acetylation levels of target genes. AtHD2A, a member of the HD2 subfamily of HDAC proteins, is highly expressed in flowers and siliques. Silencing of *AtHD2A* expression aborts seed development (Wu et al., 2000). Similarly, the *Athda7-2* mutant causes both degeneration of micropilar nuclei at the four-nucleate embryo sac stage and an overall delay of embryo development, ultimately decreasing seed fertility (Cigliano et al., 2013). Meanwhile, OsSRT1, a NAD + -dependent HDAC in rice, represses the expression of *RICE STARCH REGULATOR1* (*RSR1*) and amylase genes, thus maintaining starch accumulation in developing seeds (Zhang et al., 2016). In maize, increases in acetylated histones H3 and H4 accompanied by decreases in H3K9me2 are observed in *hda108* mutants, resulting in a wide range of plant damage, including impaired fertility of cobs (Forestan et al., 2018).

As a nutritional supply tissue, the endosperm is indispensable for seed development. Disordered timing of endosperm development leads to seed failure in interploid and interspecific hybrids, directly impeding crop breeding (Walia et al., 2009; Ishikawa et al., 2011; Kradolfer et al., 2013; Sekine et al., 2013). During cell proliferation, the PRC2 complex adds the repressive mark H3K27me3 on endosperm-related transcripts. In Arabidopsis, mutations of genes encoding members of the FERTILIZATION-INDEPENDENT SEED (FIS)–PRC2 complex [*FERTILIZATION-INDEPENDENT ENDOSPERM* (*FIE*), *MEDEA* (*MEA*), *FIS2*, *MULTICOPY SUPRESSOR OF IRA1 (MSI1)*] decrease H3K27me3 accumulation and impair cellularization of endosperm, and the mutants are characterized by a gametophytic maternal effect. After fertilization, embryonic cell proliferation and morphogenesis are inhibited in these mutants, decreasing their seed-setting rate. Notably, endosperm proliferation can also initiate in mutants in the absence of fertilization, but with arrested embryo development, producing non-functional autonomous seeds (Chaudhury et al., 1997; Grossniklaus et al., 1998; Kiyosue et al., 1999; Ohad et al., 1999; Yadegari et al., 2000; Sørensen et al., 2001; Köhler et al., 2003a,b). The downstream genes regulated by the FIS–PRC2 complex include type I MADS-box transcription factor genes, which encode key regulators in endosperm formation (Kang et al., 2008; Hehenberger et al., 2012; Figueiredo et al., 2015; Zhang et al., 2018). Moreover, the seed-abortion phenotype of a *mea* mutant can be alleviated by reducing the expression of the type I MADS-box gene *PHERES1* (*PHE1*) (Köhler et al., 2003b). Similarly, maternal loss of *AGAMOUS-LIKE62* (*AGL62*) can also rescue delayed cellularization of endosperm cells, normalizing seed development in a *fis2* mutant (Hehenberger et al., 2012). Aside from the FIS–PRC2 complex, SWINGER (SWN), a subunit of the EMBRYONIC FLOWER 1 (EMF)–PRC2 complex also participates in the initiation of endosperm development. Although a *swn* mutant shows no identifiable developmental defect, a *swn mea* double mutant has an enhanced autonomous seed formation phenotype compared to the *mea* single mutant, indicating that SWN and MEA work redundantly (Wang et al., 2006).

In cereal, the PRC2 complex is evolutionarily conserved, but shows differences in combination of subunits and in specific function compared with that in Arabidopsis, as exemplified by the absence of some homologs of Arabidopsis *FIS* genes (*MEA* and *FIS2*) in cereal genomes (Rossi et al., 2001; Springer et al., 2002; Danilevskaya et al., 2003; Spillane et al., 2007; Luo et al., 2009; Rodrigues et al., 2010). Rice has two *FIE* homologs, *OsFIE1* and *OsFIE2*, with different expression patterns: *OsFIE1* is specifically expressed in endosperm, whereas *OsFIE2* is expressed in all tissues tested. In earlier research, no autonomous endosperm development was observed in *OsFIE1* and *OsFIE2* loss-of-function plants with emasculated florets (Luo et al., 2009; Nallamilli et al., 2013). However, other studies revealed that the autonomous endosperm phenotype could be occasionally detected in unfertilized lines with *OsFIE2* defect, along with impaired cellularization, suggesting that *OsFIE2* may retain functional similarity to its Arabidopsis homolog (Li et al., 2014; Cheng et al., 2020).

Moreover, rice also has two homologs of the Arabidopsis *PcG* gene *EMBRYONIC FLOWER2* (*EMF2*): *EMF2a* and *EMF2b*. Arabidopsis EMF2 is required to maintain vegetative development in Arabidopsis (Moon et al., 2003). Rice EMF2a, a maternally expressed gene in the endosperm, is indispensable for early seed development; delayed cellularization of endosperm and subsequent autonomous endosperm is observed in emasculated spikelets of an *osemf2a* mutant. Not surprisingly, the loss of OsEMF2a function reduces H3K27me3 modifications at various type I MADS-box genes, several of which (e.g., *OsMADS77* and *OsMADS89*) may control the timing of cellularization in endosperm in a similar manner to *AGL62* and *PHE1* in Arabidopsis (Luo et al., 2009; Cheng et al., 2021; Tonosaki et al., 2021).

Seed size and weight are crucial agronomical traits, tightly linked with grain yield in crop breeding (Sweeney and McCouch, 2007). In Arabidopsis, the inner space of mature seed is mostly occupied by the embryo, and nutrients are mainly stored in the cotyledons (Zhou et al., 2013). Mutation of *EARLY FLOWERING IN SHORT DAYS* (*EFS*), also called *SDG8*, encoding the major contributor to H3K36 methylation, leads to the formation of larger embryos, resulting in enlarged seeds in Arabidopsis (Cheng et al., 2018). Similarly, larger and heavier seeds are also observed in *clf-28* lines (mutants of *CURLY LEAF* [*CLF*], which encodes the core unit of the PRC2 complex), along with a large-scale, dynamic change in H3K27me3 level during embryonic development (Liu J. et al., 2016). Unlike in dicotyledons, endosperm in monocotyledon crops is not gradually consumed during seed maturation, but filled with large amounts of starch and nutrients for storage (Olsen, 2004; Agarwal et al., 2011; Zhou et al., 2013). In rice, unlike in the Arabidopsis *clf-28* mutant, which has enlarged seeds, *OsSDG711* (*OsCLF*) downregulation lines have smaller seeds, accompanied by altered expression of starch-related genes (Liu et al., 2021). A similar phenotype of smaller seed size and reduced contents of multiple storage proteins is also observed in reduction lines of *OsFIE2* or *OsFIE1* (Nallamilli et al., 2013; Huang et al., 2016; Liu X. et al., 2016). However, overexpression of *SDG711* or *OsFIE1* also decrease seed size, but the regulatory mechanisms are not fully elucidated (Folsom et al., 2014; Liu et al., 2021). Another tissue with important effects on grain size and weight is the spikelet hull. In rice, the grain is physically restricted by the size of the hull. The quantitative trait locus (QTL) *GRAIN WEIGHT ON CHROMOSOME 6* (*GW6a*), also called *OsglHAT1*, encodes a histone acetyltransferase that regulates grain weight, hull size, yield, and plant biomass. Elevated *GW6a* expression enhances grain yield by enlarging spikelet hulls *via* increasing cell number and accelerating grain filling (Song et al., 2015).

In general, the epigenetic regulation of major seed traits in two model plants, Arabidopsis and rice, partially overlaps but also shows some divergence. The comparison of epigenetic machinery in seed development is also extended to other crop plants, like soybean and maize (Lu et al., 2015; Lin et al., 2017). Therefore, the similarity and divergence highlight the need for more knowledge of the complex network of epigenetic regulation influencing seed development programs in different species.

## Epigenetic Regulation of a Critical Seed Dormancy Pathway

Seed dormancy is an innate state in which the seed is unable to germinate, even under favorable conditions. Entry into dormancy is determined primarily by genetic factors and is also influenced by the environment surrounding the mother plant (Finch-Savage and Leubner-Metzger, 2006). Seeds in the soil seed bank (SSB) can sense seasonal signals and continually adjust their dormancy levels in order to complete germination at a suitable time of the year (Baskin and Baskin, 2006; Walck et al., 2011; Finch-Savage and Footitt, 2017).

Several histone-modification genes regulating histone ubiquitination, methylation, and acetylation exhibit dynamic expression patterns in response to seasonal change that are correlated with dormancy cycling in the SSB. Moreover, the accumulation of two antagonistic histone marks, H3K4me3 and H3K27me3, on key dormancy genes changes dynamically accompanied by changes in dormancy level, suggesting that chromatin regulators play pivotal roles in this process (Footitt et al., 2015). DOG1 is a master regulator of primary dormancy that acts in concert with ABA and HEME to delay germination (Née et al., 2017; Carrillo-Barral et al., 2020). During release from dormancy, the active H3K4me3 mark on *DOG1* chromatin is removed when the seeds are exposed to light; meanwhile, the repressive H3K27me3 mark accumulates on *DOG1* in seeds of the SSB, and light exposure amplifies this accumulation (Müller et al., 2012; Footitt et al., 2015).

The components of polycomb-group proteins, including CLF and LIKE HETEROCHROMATIN PROTEIN 1 (LHP1), are recruited by B3-domain-containing transcriptional repressors, HSI2/VAL1 (HIGH-LEVEL EXPRESSION OF SUGAR INDUCIBLE2/VIVIPAROUS-1/ABA3-LIKE1) and HSL1 (HSI2-LIKE1)/VAL2, to RY elements in the *DOG1* promoter. Hence, they accelerate the deposition of H3K27me3 marks and subsequent repression of *DOG1* expression (Chen N. et al., 2020). PICKLE (PKL) is an ATP-dependent chromatin-remodeling factor that promotes the deposition of H3K27me3 (Zhang et al., 2008, 2012). LUX ARRHYTHMO (LUX), a member of the evening complex (EC) of the circadian clock, physically interacts with PKL and recruits it to the chromatin region of *DOG1*. Correspondingly, levels of the repressive mark H3K27me3 at specific *DOG1* chromatin loci are greatly reduced in the *lux* and *pkl* mutants, increasing dormancy compared with the wild type. Moreover, these phenotypes are abolished when the mother plants are grown under continuous light. Thus, there may exist a regulatory mechanism in which EC proteins coordinate with PKL to transmit circadian signals, thereby directly regulating *DOG1* expression and seed dormancy during seed maturation (Zha et al., 2020). On the other hand, the binding site of LUX is close to the transcriptional start site of the non-coding antisense transcript *asDOG1*, which suppresses the expression of the *DOG1* sense transcript, and *asDOG1* transcription is decreased in the *pkl-1* mutant (Fedak et al., 2016; Zha et al., 2020). It suggests that the effect of PKL-LUX repression of *DOG1* transcription maybe much more elaborate than might be expected. In contrast to the H3K27me3 deposition functions of the LUX-PKL regulatory complex, RELATIVE OF EARLY FLOWERING6 (REF6), a key H3K27me3 demethylase that binds directly to the ABA catabolism genes *CYP707A1* and *CYP707A3*, is responsible for reducing their H3K27me3 levels. Correspondingly, the seeds of *ref6* mutants display enhanced dormancy, associated with increased endogenous ABA content (Chen H. et al., 2020).

Beyond H3K27me3, H3K9 and H3K4 methylation are also involved in *DOG1* and seed dormancy control. The global accumulation of H3K9 dimethylation is catalyzed by KYP/SUVH4, a Su(var)-type methyltransferase. Mutations in the *KYP* increase *DOG1* and *ABI3* expression, promoting seed dormancy. On the other hand, the sensitively of seed germination to ABA and paclobutrazol (PAC) is also increased in *kyp-2* mutant (Zheng et al., 2012). *SUVH5*, a homolog of *SUVH4*, interacts with the histone deacetylase HDA19 *in vivo* and *in vitro*. Mutants of both *SUVH5* and *HDA19* increase histone H3 acetylation (H3ac) but decrease H3K9me2, therefore enhancing *DOG1* expression and seed dormancy (Zhou et al., 2020). LYSINE-SPECIFIC DEMETHYLASE 1-LIKE1 (LDL1) and LDL2, two Arabidopsis histone demethylases, reduce the level of the histone H3-Lys 4 methylation in chromatin. They act redundantly to repress genes related to seed dormancy, including *DOG1*, *ABA2*, and *ABI3*, and *LDL1* or *LDL2* overexpression lines cause reduced seed dormancy (Zhao et al., 2015).

In yeast, H3K4me3 deposition is regulated by the SET1 histone methyltransferase (HMT) embedded in a so-called COMPlex of Proteins Associated with Set1 (COMPASS), in a process dependent on H2B monoubiquitination (H2Bub) (Miller et al., 2001; Sun and Allis, 2002). Similarly, Arabidopsis possesses homologs of all known COMPASS subunits, which potentially form several COMPASS-like complexes (Jiang et al., 2009, 2011; Fletcher, 2017). Genetic knockout of *SWD2-LIKE b* (*S2Lb*), the Arabidopsis homolog of Swd2 axillary subunit of yeast COMPASS, triggers pleiotropic developmental phenotypes, including reduced fertility and seed dormancy, accompanied by decreased H3K4me3 deposition and barely detectable *DOG1* expression (Fiorucci et al., 2019). However, even though H2B monoubiquitination regulated by HISTONE MONOUBIQUITINATION1 (HUB1) and HUB2 also increases *DOG1* expression and seed dormancy, the classical H2Bub–H3K4me3 *trans-*histone crosstalk seems to be lacking in Arabidopsis, since global H3K4me3 enrichment and the occupancy of an S2Lb-GFP (green fluorescent protein) fusion on target genes do not show obvious differences in the *hub1-3* background as compared with that in wild-type (Liu et al., 2007; Fiorucci et al., 2019).

Unlike direct regulators of histone modification, histone readers are involved in recognizing these marks and transferring the information to subsequent regulator units. The reader EARLY BOLTING IN SHORT DAYS (EBS) specifically recognizes the H3K4me2/3 mark and interacts with HDAC proteins such as HDA6 to modulate gene expression. Loss of function mutation of *EBS* reduces seed dormancy, and mutation of *EBS* homolog *SHORT LIFE* (*SHL*) deepens the seed dormancy alteration. However, EBS acts independently of two other types of dormancy regulators, HUB proteins and ARABIDOPSIS TRITHORAX-RELATED7 (ATXR7), and it does not affect the expression of *DOG1* (Liu et al., 2007, 2011; Narro-Diego et al., 2017).

Overall, studies of the epigenetic regulation of seed dormancy have mainly focused on *DOG1* expression (Figure 1B), and other regulatory pathways need further investigation to further complete the picture.

## Seed Germination in Response to Divergent Environmental Cues

Seed germination is an important physiological event that marks a transition from the quiet status of seeds to the active statues of seedlings, during which many processes are reprogrammed. The condensed chromatin state may diminish during seed germination (van Zanten et al., 2011; Zanten et al., 2013), providing a suitable environment for activating gene expression and physiological metabolisms that facilitate the process. Seeds can adjust their germination strategies in response to external environmental cues. In this section, we highlight current knowledge of the roles of histone modifiers and chromatin remodelers in regulating seed germination in response to light, temperature, and abiotic stresses (Figure 1C).

Light and temperature are two main exogenous factors determining plant growth, development, and productivity, including seed germination. In this phase, one of the classical light signal transport chains is the PHYTOCHROME B (PHYB)–PHYTOCHROME INTERACTING FACTOR1 (PIF1)/PIL5 pathway. In Arabidopsis, PHYB destabilizes PIF1 to regulate light-responses seed germination through affecting genes expression in ABA and GA pathways. *SOMNUS* (*SOM*) is an important PIF1 downstream target that negatively regulates seed germination (Oh et al., 2006; Kim et al., 2008; Luo et al., 2014; Vaistij et al., 2018). Chromatin remodeling and histone modification that participate in light-regulated seed germination mainly function by affecting this PHYB-dependent pathway.

SUVH5, an H3K9 methyltransferase, acts as a positive regulator of PHYB-dependent seed germination in Arabidopsis. It functions by repressing the transmission of ABA signal and ABA biosynthesis, as well as suppressing the expression a family of *DOG1* genes *via* H3K9me2 in imbibed seeds (Gu et al., 2019). Mutation of *EFS/SDG8*, another HMT gene, decreases H3K36me2 and H3K36me3 levels at the *PIF1* locus, resulting in reduced *PIF1* expression in imbibed seeds (Lee et al., 2014). Meanwhile, HDA15 can be recruited by PIF1 and to form a repression module that regulates light-dependent seed germination by decreasing histone H3 acetylation levels and the corresponding transcription of light-responsive genes (Gu et al., 2017). JMJ20 and JMJ22, two histone demethylation enzymes, act redundantly as positive regulators of seed germination. When PHYB is inactive, JMJ20 and JMJ22 are directly suppressed by the zinc-finger protein SOM, and the repression will be released upon PHYB activation by light. Derepressed JMJ20/JMJ22 increase seed germination rate through the removal of repressive histone arginine methylations at *GIBBERELLIN 3-OXIDASE1* (*GA3ox1*) and *GA3ox2* (Cho et al., 2012). Therefore, light treatment promotes seed germination in a process that may be partially regulated by the PHYB–PIF1–SOM–JMJ20/JMJ22 pathway.

Moreover, another JmjC-domain demethylase JMJ17 participates in ABA response in seed germination through co-regulation with WRKY DNA-BINDING PROTEIN 40 (WRKY40), HYPOCOTYL5 (HY5), and ABI5. An elevated level of H3K4me3 at *ABI5* has been detected in *jmj17* and *wrky40* mutants. In the presence of ABA, WRKY40 and JMJ17 are released from ABI5 chromatin, which allows HY5 to induce ABI5 expression. Because HY5 is another crucial factor that helps promote photomorphogenesis, the transcriptional switch composed of JMJ17–WRKY40 and HY5–ABI5 modules may play an essential role in the integration of light and ABA signaling (Wang et al., 2021).

Temperature is another critical environmental cue affecting seed germination (Toh et al., 2008). SOM participates in thermoinhibition of seed germination by altering ABA and GA metabolism (Park et al., 2011; Lim et al., 2013; Chang et al., 2018). EBS, the histone mark reader, can be recruited by AGL67 to the *SOM* locus, thus recognizing H3K4me3 at the *SOM* promoter. Under high temperature (HT), the AGL67–EBS complex is highly enriched around the *SOM* promoter, leading to deposition of the activation mark H4K5 acetylation on *SOM* and ultimately inhibiting seed germination (Li et al., 2020). POWERDRESS (PWR), a protein with a SANT-domain, interacts with ABI3 and HDAC proteins to modify histone acetylation status and the level of nucleosome histone H2A.Z incorporation in the target loci. The complex inhibits *SOM* expression by reducing H4 acetylation deposition and increasing nucleosome H2A.Z content at the *SOM* locus, thus promoting the thermotolerance of seed germination. Under HT, the *PWR* transcript decreased, resulting in releasing of *SOM* from repression state (Yang et al., 2019).

ABA, as a barrier to germination, plays a pivotal role in plant response to abiotic stresses, such as drought and salt (Zhu, 2016). Members in the SWITCH2(SWI2)/SNF2 chromatin-remodeling complexes affect seed germination under ABA treatment. BRAHMA (BRM), the core SWI2/SNF2 ATPases within the complex, directly repress the expression of *ABI5*, the *brm-3* mutant presents ABA hypersensitivity in seed germination (Han et al., 2012). SWITCH SUBUNIT3 (SWI3) proteins (called SWI3A–D) in Arabidopsis are also important subunits of SWI2/SNF2-dependent chromatin-remodeling complexes (Sarnowski et al., 2005). BRM and SWI3C show strong direct physical interaction and null *swi3c-2* mutants show an ABA-hypersensitive phenotype similar to *brm*. These observations suggest that SWI2C may be a dedicated BRM complex component (Hurtado et al., 2006; Han et al., 2012). In contrast, mutants of *SWI3B* show reduced sensitivity to ABA-mediated inhibition of seed germination, with reduced expression of the ABA-responsive genes *RAB18* and *RD29B* (Saez et al., 2008). Furthermore, in overexpression lines of *AtCHR12* or *AtCHR23*, another two SWI2/SNF2 ATPase genes, the phenotype of reduced germination is pronounced under ABA and NaCl treatment, coinciding with increased transcription of seed maturation genes (Leeggangers et al., 2015).

In addition to chromatin remodelers, HDAC proteins are also involved in abiotic-stress-responsive seed germination. In Arabidopsis, germination of the *hd2c* mutant is restrained under ABA and salinity stress, while the *hd2a* mutant is insensitive to ABA (Colville et al., 2011). HD2C interacts with HDA6 and binds to histone H3. The expressions of *ABI1* and *ABI2* are decreased along with increased H3K9K14Ac and decreased H3K9me2 modification in *hda6*, *hd2c*, and *hda6 hd2c-1* (Luo et al., 2012). Moreover, HDA6 and HDA19 may play redundant roles in modulating seed germination response to abiotic stress by increasing the expression of ABA- and abiotic-stress-responsive genes (Chen and Wu, 2010; Chen L. T. et al., 2010). Arabidopsis HISTONE DEACETYLATION COMPLEX1 (HDC1) is the rate-limiting component of the histone deacetylation complex that physically interacts with HDAC proteins to desensitize plant germination to salt, mannitol, ABA, and PAC treatments (Perrella et al., 2013). By contrast, *hda9-1* and *hda9-2* mutants show increased germination in response to ABA treatment and HDA9 forms a complex with ABI4 to regulate the expression of the ABA catabolic genes *CYP707A1* and *CYP707A2* (Baek et al., 2020). In rice, plants overexpressing *HDA705* or *HDT701* show not only delayed germination under ABA, NaCl, or polyethylene glycol (PEG) treatment, but also stronger resistance to drought stress as seedlings (Zhao et al., 2014, 2016).

## Epigenetic Factors Acting in Seed-To-Seedling Transition

After seeds germination, plants undergo an irreversible transition from embryo to seedling development, accompanied by repression of embryonic traits and emergence of vegetative tissue. Expression change of the seed-maturation genes collectively known as LAFL is important for the switch of the developmental program. In loss-of-function mutants of these genes, embryos skip late-embryonic development and enter the vegetative program prematurely (Keith et al., 1994; West et al., 1994; Nambara et al., 2000). However, when some of these genes are misexpressed in vegetative tissues, abnormally developed seedlings emerge that show induced ectopic deposition of seed storage proteins and even somatic embryo or callus formation (Parcy and Giraudat, 1997; Lotan et al., 1998; Stone et al., 2001; Gazzarrini et al., 2004; Braybrook et al., 2006; Yang et al., 2013). The factors involved in chromatin remodeling and histone modification protect normal seedling morphology mainly by repressing the transcription of LAFL genes (Figure 1D).

In the PRC1 complex, two types of ring-finger proteins, AtRING1s and AtBMI1s, are major subunits that directly catalyze H2A monoubiquitination (H2Aub). The mutants *Atring1a Atring1b* and *Atbmi1a Atbmi1b* show ectopic expression of seed-maturation genes and indeterminate embryonic traits at the seedling stage (Bratzel et al., 2010; Chen D. et al., 2010; Yang et al., 2013). Moreover, mutations of *EMF1* or *EMF2*, two PcG proteins, can strengthen the phenotype of *Atbmi1a Atbmi1b* and expression of the seed maturation genes increased obviously in the *emf1* mutant, suggesting these regulators may collaborate in repression of the maturation program after germination (Moon et al., 2003; Kim et al., 2012; Yang et al., 2013). VAL proteins are B3-type transcription factors that interact with AtBMI1 proteins. *val1/2* seedlings display phenotypic defects similar to those of *Atbmi1a/b/c* mutants, accompanied by strongly reduced H2Aub levels at seed-maturation genes and concomitant derepressed gene transcription (Suzuki et al., 2007; Yang et al., 2013). On the other hand, the levels of H3K27me3 at *LEC1*, *FUS3*, and *ABI3* are also strongly decreased in *val1/2* and *Atbmi1a/b/c* mutants (Yang et al., 2013). VAL proteins can recruit the PRC2 subunit CLF and promote the placement of H3K27me3 on target loci (Chen N. et al., 2020; Yuan et al., 2021), indicative of genetic and physical interaction between the PRC1 and PRC2 complexes.

The Arabidopsis PRC2 complex, which catalyzes H3K27me3 addition, represses seed maturation genes, as evidenced by somatic embryo emergence in vegetative tissues of double mutants deficient in redundant PRC2 subunits, i.e., CLF and SWN or EMF2 and VERNALIZATION2 (VRN2) (Chanvivattana et al., 2004; Schubert et al., 2005; Makarevich et al., 2006; Yang et al., 2013). A single mutant of Arabidopsis FIE also gives rise to the degeneration of vegetative cells into neoplastic, callus-like structures in seedlings with abolished H3K27me3 deposition (Kinoshita et al., 2001; Bouyer et al., 2011). It should be noted that EMF2 or FIE are mainly worked in repressing flower formation upon germination, though discussion of this function is outside the scope of this manuscript (Kinoshita et al., 2001; Moon et al., 2003). Furthermore, the chromatin remodeler PKL, which has a potential role in the retention of H3K27me3, acts throughout the seedling, repressing embryonic traits. Loss of PKL function reduces levels of H3K27me3 and ectopic expression of the embryo-specific genes *LEC1*, *LEC2*, and *FUS3*, resulting in seedlings with swollen primary roots, referred to as pickle roots (Ogas et al., 1997, 1999; Dean Rider et al., 2003; Henderson et al., 2004; Li et al., 2005; Carter et al., 2018).

Therefore, the PRC1 and PRC2 repressive system are tightly integrated in the transition from seed to seedling. Moreover, many other chromatin regulators also participate in this process, and some show crosstalk with the PcG working program.

In Arabidopsis, trithorax group (trxG) proteins catalyze H3K4 methylation, which play roles opposite to that of H3K27me3. Correspondingly, the trxG members (ATX1) and ULTRAPETALA1 (ULT1) counteract the effect of CLF in floral repression (Carles and Fletcher, 2009; Alvarez-Venegas, 2010). However, removal of either or both *ATX1* and *ULT1* fails to rescue the defects exhibited by an *emf1* mutant, but promote H3K27me3, causing a swollen, pickle-like root phenotype in seedlings of *emf1 atx1 ult1* triple mutants. Yeast two-hybrid assays reveal that ULT1 physically interacts with ATX1 and EMF1, and both ATX1 and ULT1 are able to bind the chromatin of seed genes, including *LEC2* and *ABI3* (Xu et al., 2018). This suggests a new, more complex framework whereby trxG acts in concert with PcG to maintain chromatin integrity and prevent seed maturation gene expression after germination. A mutation of *SDG8/EFS*, encoding an HMT that mainly mediates H3K36 methylation, acts synergistically with *emf2* to induce the deposition of the active mark H3K4me3 on seed-maturation loci, leading to the emergence of embryonic traits (Tang et al., 2012). However, the mechanism and the specific pathway whereby the activating H3K4me3 marks are deposited in the *sdg8 emf2* double mutant is still unclear.

Arrested growth and the formation of embryo-like structures on vegetative tissues can also be observed in a *hda6 hda19* double mutant. Moreover, the disturbed cell fate seen in the *hda6* mutant upon treatment with the HDAC inhibitor trichostatin A (TSA) is rescued by mutations of *lec1*, *fus3*, and *abi3*, indicating that acetylation also has effects at the seedling development stage (Tanaka et al., 2008). In animals, CHD3 chromatin remodelers are components of RPD3-containing HDAC complexes (Tong et al., 1998; Zhang et al., 1998; Fukaki et al., 2006). Therefore, whether there are connections between the HDACs and the CHD3 protein PKL that influence the repression of embryonic properties is a question in need of further study.

## Conclusion and Future Perspectives

As plants progress from seed development through seedling establishment, gene expression is dynamically affected by histone modifications and chromatin states. These epigenetic regulations include a combination of synergistic and antagonistic crosstalk between histone-modifying enzymes through specific connecting factors. By screening and analyzing regulators in the nodes of the epigenetic network, it is possible to uncover the comprehensive changes in the epigenetic modifications of pivotal genes during development.

Additionally, loss of function of epigenetic regulatory genes often gives rise to pleiotropic effects, with changes in chromatin state at the whole-genome level. To some extent, these extensive effects are mediated by various specific co-regulators that participate in diverse biological processes. Further studies to identify the working partners of epigenetic regulatory proteins will thus provide further information about the processes governing specific pathways at the epigenetic level.

Furthermore, gene transcription regulation mediated by histone modifiers or chromatin remodelers might be the later step in the cascades of environmental signal transition. Specific knowledge about how the epigenetic regulators receive these signals needs to be uncovered.

Finally, investigations to date of the effects of histone modification and chromatin remodeling on seed developmental programs have mainly focused on the model plant Arabidopsis, with few studies performed in commercial species, such as grain, fruit, and vegetable crop species. Therefore, research on classical epigenetic regulatory pathways and associated components in model plants need to be extended to other, economically important species to accelerate its application to molecular breeding for agricultural production. Moreover, better understanding of the conserved and diverse regulatory mechanisms acting in different plant species will enhance knowledge of the complex epigenetic regulatory mechanisms controlling this process.

## Author Contributions

WJ: writing-original draft. YX, XD, and XJ: review and editing. YX: supervision. All authors contributed to the article and approved the submitted version.

## Conflict of Interest

The authors declare that the research was conducted in the absence of any commercial or financial relationships that could be construed as a potential conflict of interest.

## Publisher’s Note

All claims expressed in this article are solely those of the authors and do not necessarily represent those of their affiliated organizations, or those of the publisher, the editors and the reviewers. Any product that may be evaluated in this article, or claim that may be made by its manufacturer, is not guaranteed or endorsed by the publisher.
